# Improvement of Unsaturated Fatty Acid Production from *Porphyridium cruentum* Using a Two-Phase Culture System in a Photobioreactor with Light-Emitting Diodes (LEDs)

**DOI:** 10.4014/jmb.2011.11004

**Published:** 2020-12-07

**Authors:** So Hee Kim, Ui Hun Lee, Sang Baek Lee, Gwi-Taek Jeong, Sung-Koo Kim

**Affiliations:** School of Marine, Fisheries and Life Science, Pukyong National University, Busan 48513, Republic of Korea

**Keywords:** Light-emitting diodes (LEDs), unsaturated fatty acids, photobioreactor, *Porphyridium cruentum*, eicosapentaenoic acid

## Abstract

In this study, the culture conditions for *Porphyridium cruentum* were optimized to obtain the maximum biomass and lipid productions. The eicosapentaenoic acid content was increased by pH optimization. *P. cruentum* was cultured with modified F/2 medium in 14-L photobioreactors using a two-phase culture system, in which the green (520 nm) and red (625 nm) light-emitting diodes (LEDs) were used during the first and second phases for biomass production and lipid production, respectively. Various parameters, including aeration rate, light intensity, photoperiod, and pH were optimized. The maximum biomass concentration of 0.91 g dcw/l was obtained with an aeration rate of 0.75 vvm, a light intensity of 300 μmol m^-2^s^-1^, and a photoperiod of 24:0 h. The maximum lipid production of 51.8% (w/w) was obtained with a light intensity of 400 μmol m^-2^s^-1^ and a photoperiod of 18:6 h. Additionally, the eicosapentaenoic acid and unsaturated fatty acid contents reached 30.6% to 56.2% at pH 6.0.

## Introduction

Unsaturated fatty acids are the essential components of higher eukaryotic cells [1]. The unsaturated fatty acid omega-3 (ω-3) consists of α-linolenic acid (C_18_H_30_O_2_, ALA), eicosapentaenoic acid (C_20_H_30_O_2_, EPA), and docosahexaenoic acid (C_22_H_32_O_2_, DHA). ALA can lower blood cholesterol levels and thus reduce the risk of cardiovascular diseases and protect arteries [2]. EPA can reduce blood cholesterol and triglyceride levels to suppress blood clot formation and stimulate brain function. EPA has been shown to prevent diseases such as arteriosclerosis and lung disease [3]. DHA is one of the major components of the cell membrane and is primarily found in the brain and retina. Indeed, it is one of the important components of the brain [4]. Currently, the major source of both EPA and DHA is marine fish, such as mullet, salmon, and mackerel [5]. However, overfishing has exponentially increased since the 1980s worldwide. Therefore, the use of fish as the primary source of unsaturated fatty acids is no longer a sustainable option [6]. As a result, aquaculture has become one of the fastest growing food industries in the world [7]. Soybean meal, rapeseed, sunflower seeds and corn are used as feed for aquaculture, however, they are not suitable for this purpose because they are food crops in limited supply. Thus, microalgae have potential as a sustainable and economical feed source [8, 9]. Consequently, microalgae have increasingly attracted attention due to their EPA and DHA contents.

Microalgae are primary producers found in most oceans. They use light energy and carbon dioxide to convert biomass into carbohydrates, proteins, and lipids [10]. Biomasses can be used as biofuel, food, and feed resources [11]. In addition, microalgae absorb carbon dioxide 10–50 times faster than land plants and can help solve environmental problems such as global warming [12]. Here, we investigated the microalga *Porphyridium cruentum* due to its high lipid content per dry cell weight (30–40%), rapid proliferation, and high percentage of ω-3 fatty acids [6]. Most microalga strains have been isolated and cultured under controlled conditions for efficient cultivation. Photobioreactors (PBRs) can adjust the culture conditions for microalga strains and control the metabolism of microalgae [13]. Moreover, bioreactors with light-emitting diodes (LEDs) have been used for the efficient cultivation of microalgae. However, it is not clear whether the LED chip can supply enough light energy to penetrate the 20-mm thick bioreactor wall and enter into the chamber [14, 15]. In addition, when the cell concentration is high, it is difficult to transmit light to the inside of the reactor using an external light source. To solve this issue, we used a photobioreactor equipped with an internal light source. Optimization of the LED light intensity, photoperiod, and wavelength improved lipid production and microalgae growth [16]. According to previous studies, *P. cruentum* reaches the highest biomass at the green LED wavelength (520 nm). The phycobiliproteins of *P. cruentum* are composed of phycoerythrin, phycocyanin, and small amounts of allophycocyanin. Phycoerythrin efficiently absorbs light in the green LED wavelength (520 nm) to reach high biomass [17].

In this study, we employed a two-phase culture system involving 14-L photobioreactors (PBRs) to cultivate *P. cruentum*. In the first phase, we used a green (520 nm) LED to increase the biomass production, and then the LED wavelength was switched to red (625 nm) during the second phase to stimulate lipid production. The optimization of the aeration rate, light intensity, and photoperiod increased the biomass production, while the optimization of the light intensity, photoperiod, and pH increased the lipid production. Finally, we evaluated the effect of pH on the production of ω-3 fatty acids.

## Materials and Methods

### Microalgal Strain and Culture Conditions

*P. cruentum* was obtained from the Korea Institute of Ocean Science & Technology (Kiost, Korea) and pre-cultured for 10 days in sterilized seawater with modified F/2 medium containing 75 mg NaNO_3_, 5 mg NaH_2_PO_4_·H_2_O, 4.36 mg Na_2_EDTA, 3.15 mg FeCl_3_·6H_2_O, 0.02 mg MnCl_2_·4H_2_O, 0.02 mg ZnSO_4_·7H_2_O, 0.01 mg CoCl_2_·6H_2_O, 0.01 mg CuSO_4_·5H_2_O, 0.006 mg Na_2_MoO_4_·2H_2_O, 30 mg Na_2_SiO_3_, 0.2 mg thiamine-HCl, 0.01 mg vitamin B_12_, and 0.1 mg biotin per liter [18]. The experiment was carried out with a working volume of 10 L in 14-L PBRs at 20°C and 150 rpm. The initial cell density was 1 × 10^5^ cells/ml. The cultivation process was aimed at maximum biomass production in the first phase of the culture, and maximum lipid production and polyunsaturated fatty acid (PUFA) production in the second phase.

### PBR and Illumination System

**Fig. 1:**
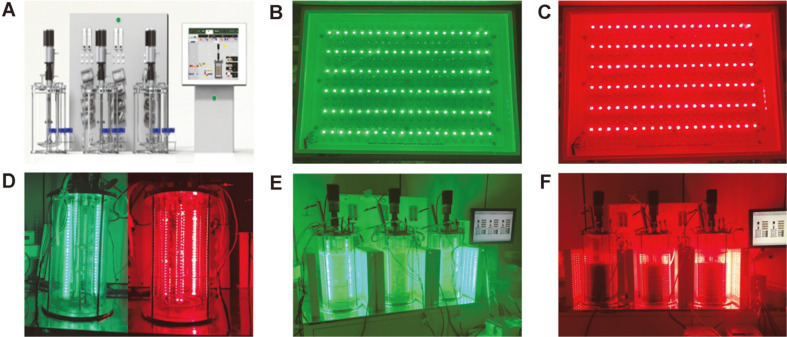
Application of three 14-L photo-bioreactors. (**A**) Graphical photographs of the 14-L photo-bioreactors. Photographs of the microalgae grown with external (**B**) green (520 nm) and (**C**) red LED (625 nm) panels and (**D**) internal LED light during the (**E**) first phase under the green LED wavelength for biomass culture and the (**F**) second phase under the red LED wavelength for lipid accumulation.

Each PBR (FMT ST series, Fermentec Co. Ltd., Korea) was made of Pyrex glass with an internal diameter and height of 200 mm and 450 mm, respectively, as shown in Fig. 1A. Filtered air was supplied through a ring sparger at the bottom of the tank, and the aeration rate was controlled by a rotameter. The agitation system consisted of 2-disk turbine impellers and a foam breaker. To enhance mixing, three baffles were set at the bottom of the PBR. The external LED panels used in this experiment had a size of 28.5 × 38.6 × 4.4 cm^3^ (Luxpia Co. Ltd., Korea) and were arranged in strips as shown in Figs. 1B and 1C. Each LED strip comprised 20 diodes spaced at 1-cm intervals vertically and horizontally. Additionally, considering the light transmittance, an LED strip composed of 30 diodes spaced vertically 1-cm apart was installed in the photobioreactor as shown in Fig. 1D. The green LED wavelength (520 nm) was used during the first phase for biomass production (Fig. 1E), and the red LED wavelength (625 nm) during the second phase for lipid production (Fig. 1F). Light intensity was measured at the center of the PBR by a light sensor (HD2102.2; Ohm S.R.L., Italy).

### Experimental Design

A two-phase culture system was used to enhance the biomass, lipid, and PUFA yields from *P. cruentum*. Toward this end, culture conditions were separately optimized for each of the two phases. In the first phase, various growth conditions, including the aeration rate (0.25, 0.50, and 0.75 vessel volumes per minute; vvm), light intensity (100, 200, 300, 400, 500, and 600 μmol m^-2^s^-1^), and photoperiod cycle (12:12, 18:6, and 24:0 h) based on the green LED wavelength (520 nm) were evaluated for the maximum cell growth. After determining the optimal aeration rate, light intensity, and photoperiod cycle for the cell growth in the first phase of the culture, the conditions for the lipid production were optimized in the second phase by controlling the light intensity (100, 200, 300, 400, 500, and 600 μmol m^-2^s^-1^), pH (5.0, 6.0, 7.0, 8.0, and 9.0), and photoperiod cycle (12:12, 18:6, and 24:0 h) using the red LED wavelength (625 nm). The aeration rate, light intensity, and photoperiod were optimized to carry out the first phase culture until day 7. The second phase culture was then carried out from day 8 onward at pH 5.0 using the citrate buffer, at pH 6.0, 7.0, or 8.0 using the potassium phosphate buffer, and at pH 9.0 using the tris(hydroxymethyl) aminomethane buffer as previously described [19]. Untreated cultures were used as controls. The end of the first phase was defined as the point at which the cells reached the stationary phase due to the depletion of the nitrogen source. When the cell growth reaches the stationary phase, the second phase is initiated to produce lipids.

### Assessment of Microalgal Growth

Dry cell weight was determined by an ultraviolet–visible spectrophotometer (Ultrospec 6300 Pro; Biochrom Ltd., UK) at an optical density of 540 nm (OD_540_) [20].

The correlation equation (R^2^ = 0.99) for the dry cell weight of *P. cruentum* at OD_540_ is shown below in Eq. (1):

(1) $$\mathrm{Dry}\mathrm{cell}\mathrm{weight}(\mathrm{dcw})\mathrm{of}P.\mathrm{cruentum}(g\mathrm{dcw}L^{-1})=0.77({\mathrm{OD}}_{540})(R^{2}=0.99)$$

### Assessment of Total Lipid Content

Cells were harvested using a centrifuge (Supra R22; Hanil Scientific Inc., Korea) at 8,000 ×*g* for 10 min. The precipitated biomass was washed with distilled water twice and then dried in a freeze dryer (SFDSM-24L; SamWon Industry, Korea). Subsequently, 5 mL of distilled water was added to 10 mg of dried cell biomass, which was then sonicated for 10 min using a sonicator (100 W, 20 kHz, 550 Sonic Dismembrator; Fisher Scientific Inc., USA). Total lipid content (% of DCW) was determined using methanol and chloroform following a modified solvent-based method [21] as shown in Eq. (2).

(2) $$\mathrm{Lipid}\mathrm{content}(\%\mathrm{of}\mathrm{dcw})=\frac{(W_{2}-W_{1})\times 100}{\mathrm{DCW}}$$

W_1_ (g) is the weight of the empty 20-ml glass tube, and W_2_ (g) is the weight of the tube with extracted lipids. DCW (g) is the dried microalgal biomass.

### Fatty Acid Methyl Ester (FAME) Measurement

The direct transesterification method reported by Dhup and Dhawan [22] was used to convert extracted lipids to FAMEs. FAMEs were then analyzed using gas chromatography (GC, YL 6100; Young Lin Inc., Korea) by employing a flame ionization detector (FID) and a silica capillary column (30 m × 0.32 mm × 0.5 μm, HP-INNOWAX; Agilent Technologies, USA). The column temperature was adjusted as follows: 140°C for 5 min, followed by an incremental temperature increase to 240°C at a rate of 5°C/min and subsequent incubation at 240°C for 10 min. The injector and FID temperatures were set at 250°C. FAMEs were identified by comparing their retention times against those of authentic standards.

### Statistical Analyses

Each experiment was conducted in triplicate. The statistical significance of the cell biomasses and lipid contents were evaluated by one-way analysis of variance (ANOVA) and Duncan’s multiple range test (*p* < 0.05) using SPSS software (ver. 23.0; SPSS Inc., USA).

## Results and Discussion

### Effect of Aeration Rate Under Green LED Wavelength

**Fig. 2:**
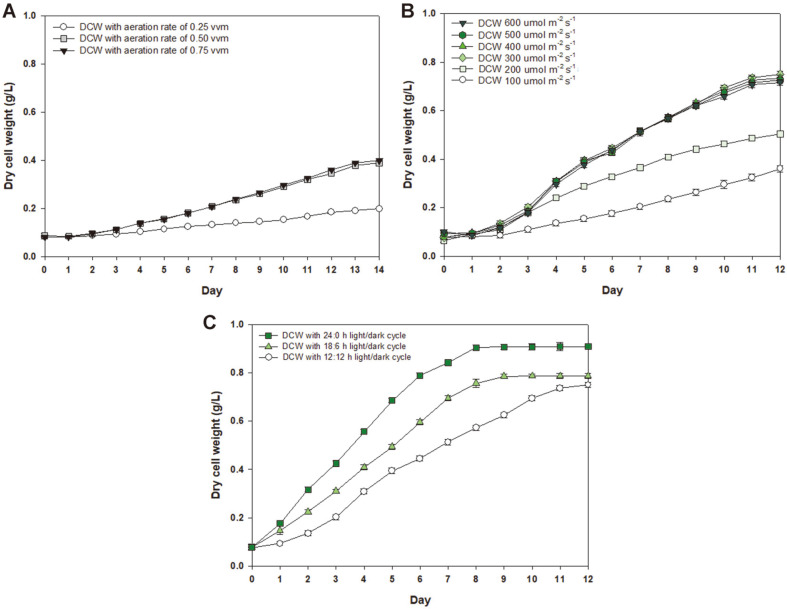
Optimization of maximum biomass production of *P. cruentum* cultured in 14-L photo-bioreactors under the green LED wavelength with various (A) aeration rates, (B) light intensities alongside 0.75 vvm aeration rate, and (C) photoperiods alongside 0.75 vvm aeration rate and 300 μmol m^-2^s^-1^ light intensity.

The photoperiod was set at a 12:12 h light/dark cycle, with a light intensity of 100 μmol m^-2^s^-1^. Following aeration rate optimization, the photoperiod and light intensity were optimized as well. Aeration rates of 0.25, 0.50, and 0.75 vvm were tested with a green LED wavelength (520 nm). Fig. 2A shows that cells with 0.75 vvm aeration rate produce the highest biomass of *P. cruentum* (0.40 g dcw/l) by day 14, followed by 0.50 vvm (0.38 g dcw/l) and then 0.25 vvm (0.20 g dcw/l). These results indicate that the CO_2_ supply should be well mixed with the culture. Microalgal biomass production is enhanced by sufficient CO_2_ supply and aeration. However, excessive aeration rates can cause shear stress to the cells, causing cell damage [23]. Sirisuk *et al*. [15] reported that increasing the aeration rate from 0.25 vvm to 0.50 vvm increases the cellular biomass from 0.33 g dcw/l to 0.38 g dcw/l in *Nannochloropsis oceanica* cultures. To obtain large microalgal biomasses, a high aeration rate and a high level of CO_2_ are required; however, the aeration rate must be limited due to the eventual cell damage [24]. The maximum biomass in this study was achieved with a 0.75 vvm aeration rate, and the CO_2_ aeration rate was set to this optimal value to obtain the largest microalgal biomass from our culture.

### Effect of Green LED Light Intensity Under Green LED Wavelength

After determining the optimal aeration rate, the effect of light intensity on biomass production was evaluated. *P. cruentum* was cultured at the optimum aeration rate of 0.75 vvm with the green LED wavelength. Light intensities of 100, 200, 300, 400, 500, and 600 μmol m^-2^s^-1^ were tested in this study. Fig. 2B shows that *P. cruentum* produced 0.75 g dcw/l of biomass by day 12 with 300 μmol m^-2^s^-1^ of light intensity, followed by 0.74, 0.73, 0.72, 0.50, and 0.40 g dcw/l of biomass with respective light intensities of 400, 500, 600, 200, and 100 μmol m^-2^s^-1^. Similarly, Wahidin *et al*. [25] reported a correlation between light intensity and microalgal biomass. *Nannochloropsis* sp. produces more biomass with 100 μmol m^-2^s^-1^ of light intensity than those with 50 or 200 μmol m^-2^s^-1^. Thus, microalgae produce large biomass amounts at high light intensities; however, when overexposed to light, cells can be inhibited by additional light. On the other hand, light intensity plays an important role in microalgae cultivation, and the requirements vary greatly depending on depth and density. When microalgae are cultured at high cell concentrations, the light intensity must be increased for light transmission. In our work, the least biomass was produced with the lowest light intensity of 100 μmol m^-2^s^-1^; however, there was no significant difference between the biomass produced at 300, 400, 500, and 600 μmol m^-2^s^-1^. Therefore, 300 μmol m^-2^s^-1^ was chosen as the optimal condition for the production of the highest biomass with the lowest power consumption.

### Effect of Photoperiod Under Green LED Wavelength

After determining the optimal aeration rate of 0.75 vvm and light intensity of 300 μmol m^-2^s^-1^, the photoperiod was optimized. Photoperiods of 12:12, 18:6, and 24:0 h light/dark cycles were used under the green LED wavelength. Fig. 2C shows that *P. cruentum* yielded the highest biomass of 0.91 g dcw/l by day 8 with 24:0 h light/dark cycle, followed by 18:6 h (0.79 g dcw/l) and then 12:12 h (0.75 g dcw/l). The biomass productivity of *P. cruentum* was 0.0625 g dcw/l/day at day 12 of culture when light intensity was optimized. However, the productivity reached 0.114 g dcw/l/day on day 8 of culture after the optimization of light intensity and photoperiod. In addition, the incubation time of the cells cultured in the 24:0 h light/dark cycle was shorter than that of the 12:12 h light/dark cycle. The stationary phase was reached by days 12, 9, and 8 with the 12:12 h, 18:6 h, and 24:0 h light/dark cycles, respectively. The growth rate of biomass changed according to photoperiod. Sirisuk *et al*. [26] have reported that *Phaedactylum tricornutum* and *Nannochloropsis salina* grow faster with a 24:0 h light/dark cycle than these same species with a 12:12 h light/dark cycle. However, *Isochrysis galbana* proliferates the most with an 18:6 h light/dark cycle comparing to 12:12, and 24:0 h light/dark cycles. Although excess light has an inhibitory effect on cell growth, *P. cruentum* was not negatively affected by the 24:0 h light/dark cycle in this study. Therefore, the 24:0 h light/dark cycle was selected to produce the highest biomass yield.

### Effect of Red LED Light Intensity in Second Phase

**Fig. 3:**
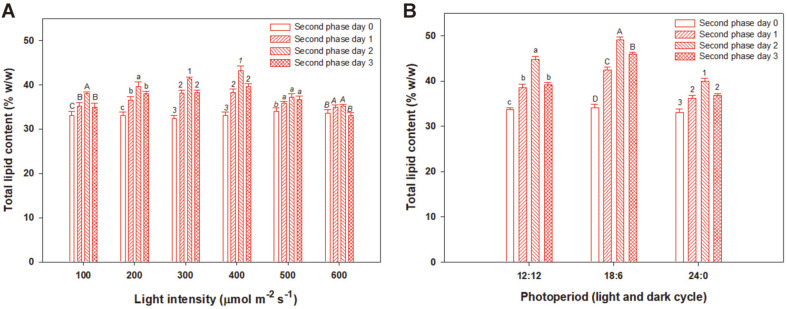
Total lipid content during the second phase of *P. cruentum* culture under various (A) light intensities and (B) photoperiods of the red LED illumination. Different letters and numbers indicate the significant differences (*p* < 0.05, Duncan’s test).

The optimal conditions for biomass production in the first phase of the culture were determined to be 0.75 vvm aeration rate, 300 μmol m^-2^s^-1^ light intensity, and a photoperiod of 24:0 h light/dark cycle under the green LED wavelength. Lipid production was induced with the red LED wavelength in the second phase. Light intensities of 100, 200, 300, 400, 500, and 600 μmol m^-2^s^-1^ were used under the red LED wavelength. The light intensity in the second phase of the culture was evaluated for 3 days to determine the optimal culture time for maximum lipid production. Fig. 3A shows that the lipid content varied based on the light intensity. *P. cruentum* showed the highest lipid production of 43.3% (w/w) (0.39 g/l) with 400 μmol m^-2^s^-1^ of light intensity. The lipid content was increased by 10.3% compared to 33.0% (w/w) (0.30 g/l) lipid on day 0 of the second phase culture with light intensity of 400 μmol m^-2^s^-1^, and the lipid productivity was 39.4 mg/l/day on day 2 of second phase. Maximum total lipid productions of 38.0, 39.6, 41.3, and 43.3% (w/w) (0.35, 0.36, 0.38, and 0.39 g/l) were obtained at light intensities of 100, 200, 300, and 400 μmol m^-2^s^-1^, respectively. However, light intensities above 500 μmol m^-2^s^-1^ suppressed lipid production. The increase of lipid production upon adjustment of the light intensity is mediated by fatty acid biosynthesis enzymes, such as acetyl CoA carboxylase and ATP/citrate lyase, which are highly active at high light-energy levels. However, the lipid biosynthesis pathway is inhibited when the light energy exceeds the saturation intensity, thus decreasing lipid production [27]. In addition, Dunstan [28] reported a direct correlation between light intensity and lipid production in microalgae and indicated that the relationship between the light intensity and photosynthesis depends on the chlorophyll type and content of microalgae.

### Effect of Photoperiod Under Red LED in Second Phase

After determining an optimal light intensity of 400 μmol m^-2^s^-1^ in the second phase of the culture, the photoperiod was optimized for the highest lipid production. Photoperiods of 12:12, 18:6, and 24:0 h light/dark cycles were used under the red LED wavelength. The microalgae were cultured for up to 3 days during the second phase to determine the optimal culture time for maximum lipid production. The lipid production of *P. cruentum* during the two-phase culture is shown in Fig. 3B. By day 2 of the second phase of the culture, *P. cruentum* generated the highest lipid production of 49.1% (w/w) (0.45 g/l) with the 18:6 h light/dark cycle, similar to the trend reported by Wahidin *et al*. [25]. The lipid content was increased by 15.1% compared to 34.0% (w/w) (0.31 g/l) lipid on day 0 of the second phase culture with 16:8 h light/dark cycle, and the lipid productivity was 44.7 mg/l/day on day 2 of second phase. *Nannochloropsis* sp. produces the highest lipid content of 31.3% with an 18:6 h light/dark cycle among 12:12, 18:6, and 24:0 h light/dark cycles. Photoperiods have been reported to induce significant changes in the total chemical compositions, pigment contents, and photosynthetic activities of microalgae [29]. The light source is an essential requirement for the production of triacylglycerides. The required light intensity and photoperiod vary from species to species [30].

### Effect of pH on Lipid Content of *P. cruentum*

**Fig. 4:**
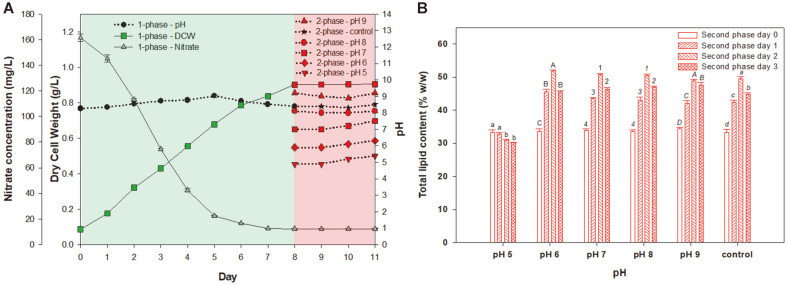
(A) Time course profile of *P. cruentum* biomass production (line) versus pH (dotted line) in the second phase. The green and red shades represent the first and second phases of the culture, respectively, and the culture pH was set to 5.0, 6.0, 7.0, 8.0, or 9.0. (B) The total lipid content during the second phase with various pH values. Different letters and numbers indicate the significant differences (*p* < 0.05, Duncan’s test).

The two-phase culture was performed following the optimization of aeration rate, light intensity, and photoperiod. *P. cruentum* was cultured under a green LED wavelength at an aeration rate of 0.75 vvm, a light intensity of 300 μmol m^-2^s^-1^, and under a 24:0 h light/dark cycle for 8 days in the first phase of the culture. The pH of the first phase was not adjusted by using the seawater. Microalgae growth under varying pH conditions showed that most marine microalgae produced the highest biomass content in pH 8.0-9.0 [31]. The seawater used in this study was between pH 8.3-9.0. Subsequently, the LED wavelength was switched from green to red light with the intensity of 400 μmol m^-2^s^-1^, and the cells were cultured with a photoperiod of 18:6 h light/dark cycle for 2 days in the second phase of the culture to obtain the highest total lipid production as shown in Fig. 4A. The control cultures were not pH-adjusted and their pH ranged from 8.3 to 8.5. Various pH values (5.0, 6.0, 7.0, 8.0, and 9.0) were tested for maximum lipid production alongside the control for 3 days in the second phase. Fig. 4B shows that *P. cruentum* produced the highest lipid contents of 51.8% (w/w) (0.47 g/l) by day 2 of the second phase at pH 6.0, followed by 50.7, 50.5, 49.5, and 48.9% (w/w) (0.46, 0.46, 0.45, and 0.44 g/l) of lipid production with pH 7.0, 8.0, control, and pH 9.0 at day 2 of the second phase, respectively. *P. cruentum* produced 18.6% more lipid on day 2 of the second phase at pH 6.0, compared to 33.6% (w/w) (0.31 g/l), which was the lipid content on day 0. In addition, lipid productivity increased from 38.2 to 47.1 mg/l/day through two-phase culture. High lipid production was obtained at pH 5.0 at 33.3% on day 0 of the second phase. However, the lipid content decreased over time at pH 5.0. The lipid content increased from 33.5% to 51.8% at pH 6.0. Lipid productivity increased from 0.0381 g dcw/l/day on day 0, to 0.0471 g dcw/l/day on day 2 of second phase culture at pH 6.0 by optimizing the light intensity, photoperiod, and pH.

The pH of the culture affects microalgal lipid production, resulting in increased lipid production under pH stress [32]. The activity of acetyl-CoA carboxylase, one of the key enzymes in lipid biosynthesis, is known as pH-dependent [33] and is inhibited at pH 5.0. In line with this data, adjusting the pH from the control pH of 8.5 to pH 6.0 increased the lipid production in this study.

### Effect of pH on Fatty Acid Composition of *P. cruentum*

The effect of the pH on the second-phase culture for the fatty acid composition of *P. cruentum* biomass was studied. The fatty acid compositions of *P. cruentum* cultured at different pHs (5.0, 6.0, 7.0, 8.0, and 9.0) alongside the control for up to 3 days are shown in Table 1. Relative to the control, there was no significant change for 3 days at pH 5.0 in the fatty acid composition (Tables 1A and 1B). However, the unsaturated fatty acid levels increased to 30.6% (144.0 mg/l) EPA and 14.5% (68.3 mg/l) DHA on day 2 at pH 6.0 and 23.5% (110 mg/l) EPA and 12.4%(58.3 mg/l) DHA on day 3 at pH 7.0. The levels of saturated fatty acids, such as stearic acid (C18:0), were decreased as shown in Tables 1C and 1D. The unsaturated fatty acid level reached to 56.2% (w/w) at pH 6.0. The total unsaturated and saturated fatty acid levels were estimated to be 56.2% (w/w) and 43.8% (w/w) as shown in Table 1C, respectively. The EPA level reached to 30.6% (w/w). At pH 7.0, the saturated fatty acid level decreased to 51.6% (w/w) as shown in Table 1D, while the unsaturated fatty acid level increased to 48.4% (w/w). In addition, comparing to day 0 of the second-phase culture, the EPA level increased from 8.5 % to 23.6% (w/w) as shown in Table 1D. When the culture pH was increased to 8.0, the total saturated fatty acid level decreased from 66.8% to 53.8% (w/w), and the unsaturated fatty acid level increased from 33.2% to 46.2% (w/w) as shown in Table 1E. Similar results have been reported for *Isochrysis galbana* at pHs 6.0, 7.0, and 8.0 [34]. *Isochrysis galbana* produces higher levels of polyunsaturated fatty acids, such as EPA, and DHA at pH 6.0 than at pH 7.0 or 8.0. Changes in pH affect the properties of the cell surface, such as adhesion onto the substrata and biomass aggregation [35]. As the pH decreases, the carboxylate ions receive protons and can be converted to neutral carboxyl groups, whereby the negative charge of the cells is neutralized and the cell dispersion stability is destroyed. This results in aggregation and sedimentation of the cells [36]. Such effects alter the permeation of ions, acids, and bases into the cell, and affect the biochemical metabolism and conformation of macromolecules, as well as the *K*_m_ values of enzymes [35]. Microalgal cell membranes are damaged by rapid pH changes. To prevent this damage, microalgae further accumulate unsaturated fatty acids to increase cell membrane fluidity. Under these cell membrane-damaging stresses, the three desaturase genes D5-desaturase, plastid acyl-ACP D9 desaturase, and microsomal D12-desaturase are upregulated to increase the unsaturated fatty acid production. However, excessive drops in pH make cell membrane maintenance impossible [37]. Therefore, the unsaturated fatty acid composition changes with the pH of the environment. *P. cruentum* limits the production of unsaturated fatty acids at pH below 6.0.

## Conclusion

According to these results, the optimal culture conditions for maximizing biomass yield during the first phase are an aeration rate of 0.75 vvm, a light intensity of 300 μmol m^-2^s^-1^, and 24:0 h photoperiod under green (520 nm) LED light. A maximum biomass production of 0.91 g dcw/l could be achieved. In the second-phase culture, we aimed to increase the lipid content, and the optimal conditions were obtained with the red LED wavelength (625 nm) at the light intensity of 400 μmol m^-2^s^-1^, under an 18:6 h photoperiod at pH 6.0. The maximum lipid content reached 51.8% (w/w) and productivity reached 47.1 mg/l/day. In addition, the EPA and DHA productivity were increased to 14.4 and 6.8 mg/l/day at pH 6.0, respectively. The unsaturated fatty acid productivity reached 26.4 mg/l/day.

## Figures and Tables

**Table 1:** Fatty acid methyl ester (FAME) contents as % of total fatty acids of *P. cruentum* during the second phase of the culture at (A) control, (B) pH 5, (C) pH 6, (D) pH 7, (E) pH 8, and (F) pH 9.

Free fatty acid (% of total fatty acid)	(**A**) Control					(**B**) pH 5					(**C**) pH 6			
				
	Day 8	Day 9	Day 10	Day 11		Day 8	Day 9	Day 10	Day 11		Day 8	Day 9	Day 10	Day 11

Myristic acid (C14:0)	0.59±0.01	0.24±0.02	0.27±0.05	0.10±01		0.82±0.05	0.68±0.08	0.92±0.02	1.05±0.01		1.08±0.01	1.84±0.01	0.11±0.01	0.11±.0.1
Palmitic acid (C16:0)	6.57±0.06	6.70±0.05	7.00±0.01	4.56±0.05		5.75±0.01	5.75±0.08	6.36±0.22	7.29±0.08		6.53±0.02	2.97±0.02	2.45±0.02	2.88±0.02
Palmitoleic acid (C16:1)	0.58±0.01	0.37±0.01	0.31±0.02	1.15±0.01		0.95±0.01	0.60±0.07	0.06±0.01	0.07±0.01		0.90±0.01	2.29±0.02	1.36±0.01	1.33±0.01
Stearic acid (C18:0)	56.84±0.19	47.82±0.15	43.91±0.21	48.48±0.25		55.77±0.22	55.32±0.05	58.60±0.02	59.61±0.22		53.24±0.02	46.81±0.25	36.24±0.26	38.96±0.26
Oleic acid (C18:1)	11.83±0.22	12.46±0.11	12.96±0.12	5.23±0.5		12.30±0.04	11.45±0.12	10.11±0.12	11.64±0.12		12.20±0.05	4.29±0.01	2.91±0.08	6.07±0.04
Linoleic acid (C18:2)	0.71±0.02	3.08±0.05	3.40±0.05	6.76±0.04		0.73±0.01	0.69±0.01	0.35±0.05	0.07±0.01		0.78±0.02	4.29±0.04	1.60±0.01	5.73±0.04
Arachicidic acid (C20:0)	0.86±0.05	0.07±0.1	0.08±0.01	0.46±0.01		0.89±0.02	0.80±0.01	1.02±0.04	1.17±0.01		0.77±0.01	0.32±0.02	0.14±0.01	0.53±0.01
Linolenic acid (C18:3)	2.36±0.17	2.77±0.3	2.89±0.06	5.06±0.01		2.46±0.03	2.72±0.12	2.93±0.01	3.42±0.06		2.35±0.02	4.85±.03	5.16±0.04	5.87±0.02
Eicosapentaenoic acid (C20:5)	**8.28±0.05**	**11.48±0.15**	**12.19±0.07**	**10.56±0.09**		**9.08±0.04**	**10.40±0.25**	**9.35±0.08**	**9.11±0.05**		**9.99±0.11**	**17.51±0.04**	**30.63±0.15**	**21.12±0.12**
Behenic acid (C22:0)	2.37±0.01	3.32±0.11	3.50±0.06	6.14±0.07		2.20±0.02	1.83±0.03	1.72±0.01	1.09±0.01		3.21±0.02	2.18±0.05	4.87±0.02	7.11±0.08
Docosahexaenoic acid (C22:6)	**9.01±0.05**	**11.70±0.03**	**13.48±0.08**	**11.50±0.12**		**9.06±0.03**	**9.78±0.19**	**8.57±0.09**	**5.47±0.11**		**8.94±0.09**	**12.65±0.14**	**14.54±0.13**	**10.28±0.18**

Saturated fatty acid	67.23	58.15	54.76	59.73		65.42	64.38	68.63	70.22		64.53	54.12	43.80	49.59
Unsaturated fatty acid	32.77	41.85	45.24	40.27		34.58	35.62	31.37	29.78		35.17	45.88	56.20	50.41

Free fatty acid (% of total fatty acid)	(**D**) pH 7					(**E**) pH 8					(**F**) pH 9			
				
	Day 8	Day 9	Day 10	Day 11		Day 8	Day 9	Day 10	Day 11		Day 8	Day 9	Day 10	Day 11

Myristic acid (C14:0)	0.61±0.01	2.03±0.01	0.19±0.01	0.83±0.05		0.63±0.01	0.15±0.01	0.17±0.01	0.09±0.01		0.51±0.01	0.30±0.01	0.18±0.01	0.13±0.01
Palmitic acid (C16:0)	6.66±0.02	3.28±0.01	2.84±0.05	4.66±0.05		6.97±0.02	5.92±0.09	6.78±0.05	4.58±0.12		6.61±0.12	6.98±0.04	7.75±0.15	4.45±0.15
Palmitoleic acid (C16:1)	0.65±0.01	2.77±0.02	1.24±0.01	1.36±0.01		0.62±.0.01	0.43±0.01	0.07±0.01	1.28±0.03		0.57±0.01	0.49±0.06	0.07±0.01	1.30±0.09
Stearic acid (C18:0)	55.30±0.28	50.2±0.20	43.46±0.18	41.07±0.19		56.04±0.19	52.60±0.32	43.20±0.19	47.24±0.26		58.98±0.31	46.42±0.19	42.90±0.22	47.98±0.22
Oleic acid (C18:1)	12.44±0.25	4.80±0.04	2.70±0.01	3.74±0.05		11.96±0.15	10.39±0.17	12.44±0.11	4.98±0.05		11.39±0.09	12.84±0.11	12.24±0.09	4.92±0.18
Linoleic acid (C18:2)	0.64±0.01	3.85±0.04	2.78±0.01	2.78±0.04		0.72±.0.05	2.43±0.05	2.90±0.08	7.13±0.09		0.67±0.01	2.84±0.13	3.07±0.09	6.91±0.19
Arachicidic acid (C20:0)	0.92±0.01	0.29±0.01	0.19±0.01	0.46±0.08		0.88±0.09	0.02±0.01	0.13±0.01	0.43±.01		0.84±0.01	0.12±±0.01	0.07±0.01	0.44±0.01
Linolenic acid (C18:3)	2.71±0.02	4.59±0.02	5.87±0.05	5.39±0.09		2.32±0.01	2.31±0.06	3.29±0.09	5.03±0.01		2.24±0.07	2.72±0.02	2.79±0.08	4.98±0.21
Eicosapentaenoic acid (C20:5)	**8.45±0.26**	**14.57±0.14**	**23.58±0.17**	**23.54±0.02**		**8.34±0.09**	**12.18±0.11**	**13.03±0.01**	**10.97±0.11**		**7.13±0.05**	**11.90±0.11**	**12.54±0.09**	**10.24±0.11**
Behenic acid (C22:0)	2.43±0.09	2.12±0.01	4.96±.0.14	3.96±0.01		2.26±0.01	2.25±0.01	3.49±0.07	5.93±0.08		2.26±0.09	3.36±0.05	3.54±0.01	6.50±0.02
Docosahexaenoic acid (C22:6)	**9.20±0.17**	**11.50±0.21**	**12.20±0.05**	**12.42±0.12**		**9.27±.0.11**	**11.31±0.09**	**14.51±0.19**	**12.34±0.16**		**8.80±0.11**	**12.02±0.19**	**14.85±0.12**	**12.15±0.34**

Saturated fatty acid	65.92	57.92	51.64	50.97		66.78	60.95	53.76	58.27		69.20	57.19	54.44	59.50
Unsaturated fatty acid	34.08	42.08	48.36	49.03		33.22	39.05	46.24	41.73		30.80	42.81	45.56	40.50
